# Distribution and Clinical Profile of Human Parainfluenza Viruses in Hospitalized Patients With Acute Febrile Illness

**DOI:** 10.1155/ijm/7072067

**Published:** 2025-11-18

**Authors:** Santhosha Devadiga, Nachiket M. Godbole, Prasad Varamballi, Chiranjay Mukhopadhyay, Anup Jayaram

**Affiliations:** Manipal Institute of Virology, Manipal Academy of Higher Education, Manipal, Karnataka, India

**Keywords:** clinical, good health, human parainfluenza virus, lower respiratory tract infection, pediatric

## Abstract

**Introduction:**

Human parainfluenza viruses (HPIVs) are significant causes of respiratory infections, particularly in children, yet their epidemiology remains poorly understood in low- and middle-income countries. HPIVs contribute to 20%–40% of pediatric lower respiratory tract infections (LRTIs) and are a leading cause of croup and hospitalizations. This study was aimed at determining the incidence, distribution, and clinical and laboratory characteristics of HPIV in hospitalized acute febrile illness (AFI) patients.

**Methods:**

A total of 12,409 AFI cases from 2016 to 2018 were tested for HPIVs via molecular methods. RNA was extracted from throat swab samples and tested via multiplex real-time RT-PCR for HPIV Serotypes 1–4. The demographic, clinical, and laboratory data of HPIV-positive patients were analyzed statistically.

**Results:**

HPIVs were detected in 217 (1.75%) patients, with HPIV-3 (49.77%) being the most prevalent, followed by HPIV-4 (18.90%), HPIV-2 (17.52%), and HPIV-1 (13.83%). HPIV-3 exhibited distinct seasonal peaks, mainly affecting children (1–9 years). Significant variations in hematological and biochemical markers were observed among serotypes and age groups. Upper and lower respiratory symptoms, along with gastrointestinal issues and systemic manifestations such as chills, myalgia, and weakness, are commonly reported.

**Conclusion:**

HPIVs contribute to respiratory illness across diverse demographics. HPIV-3 is the predominant serotype, with distinct seasonal and age-related patterns. Improved surveillance and diagnostics could aid in better management and reduce unnecessary antibiotic use.

## 1. Introduction

Human parainfluenza viruses (HPIVs) are enveloped single-stranded negative-sense RNA viruses, and there are four major serotypes (HPIVs 1, 2, 3, and 4) (1). HPIVs 1 and 3 belong to the genus *Respirovirus*, and HPIVs 2 and 4 are members of the genus *Orthorubulavirus* of the family Paramyxoviridae (2). A previous study on HPIV among children reported that 20%–40% of children were hospitalized with lower respiratory tract infection (LRTI) (3). HPIVs are the second most common cause of hospitalization in children and a common cause of croup. Infection may lead to life-threatening LRTIs, including bronchitis, bronchiolitis, and pneumonia. Reinfection is typically associated with milder disease limited to the upper respiratory tract (4).

The burden of HPIV infection among children and adults in low- and middle-income countries remains poorly understood, despite these regions being home to much of the global population. Several factors contribute to this data deficiency, including inadequate testing due to expensive molecular diagnostic assays, the ability to record HPIV-related morbidity and mortality, and the unavailability of specific treatments, which are major setbacks for HPIV diagnosis. Comprehensive data on HPIV infections could help physicians reduce unnecessary antibiotic prescriptions. HPIV infections occur worldwide, with varying seasonal patterns and circulation serotypes in different geographic regions. In India, data on the distribution and seasonality of HPIV are limited. Additionally, there are insufficient data on HPIV-associated hospitalizations for acute respiratory illness (ARI). This study is aimed at understanding the incidence of HPIVs in hospitalized acute febrile illness (AFI) patients and understanding the demographic and clinicolaboratory characteristics of HPIVs among children and adults.

## 2. Materials and Methods

### 2.1. Study Population

A total of 217 HPIV-positive cases were included in this study, and the cases represented 10 states of India: Karnataka, Kerala, Assam, Goa, Maharashtra, Jharkhand, Tripura, Tamil Nadu, and Odisha. Samples were collected as part of the AFI surveillance study conducted by the Manipal Institute of Virology (MIV), with a case definition of patients admitted to the hospital with fever (≥ 38°C) aged between 1 and 65 years (5). All the inpatients admitted to the hospitals fulfilling the inclusion criteria of the case definition were included in the surveillance study after providing informed consent. In the case of children aged 13–17 along with informed consent, ascent was also taken. The total number of AFI samples that were obtained annually from January 2016 to September 2018 is as follows: 2016–12,414, 2017–20,359, and 2018–4954. A total of 12,409 patients presented with AFI with ARI and were tested for HPIVs (2016-3641, 2017-6528, and 2018-1635, respectively). All 12,409 samples were tested for various pathogens, including viruses and bacterial agents causing AFI with ARI at MIV via molecular methods. Briefly, RNA was extracted from throat swab samples via a QIAamp Viral RNA Mini Kit (QIAGEN, Hilden, Germany) according to the manufacturer's instructions. Multiplex real-time RT-PCR was performed with primers and probes targeting HPIV Serotypes 1, 2, 3, and 4 via a Respiratory Pathogens 21 Kit (Fast Track Diagnostics [FTD], Luxembourg). RT-PCR was performed via an AgPath-ID One-step RT-PCR Kit (Applied Biosystems, Foster City, United States). The reaction was performed via a QuantStudio 5 PCR system (Applied Biosystems, Foster City, United States). A cycle threshold (CT) value of < 35 was considered positive for HPIV. Demographic, clinical, and laboratory data of HPIV-positive patients were obtained from the AFI study database after ethical clearance was obtained for analysis.

### 2.2. Data Analysis

We assessed the clinical, laboratory, and demographic data of HPIV-positive patients. For the analysis of continuous variables in two groups, a two-tailed t test was used; for more than two groups, one-way ANOVA was used, and for the analysis of categorical variables, the chi-square test was used. A *p* value of < 0.05 was set as the level of statistical significance. Statistical significance was determined by one-way ANOVA followed by Tukey's multiple comparisons test. To compare the data, they were analyzed via GraphPad Prism Version 8.4.2.

## 3. Results

**Figure 1:**
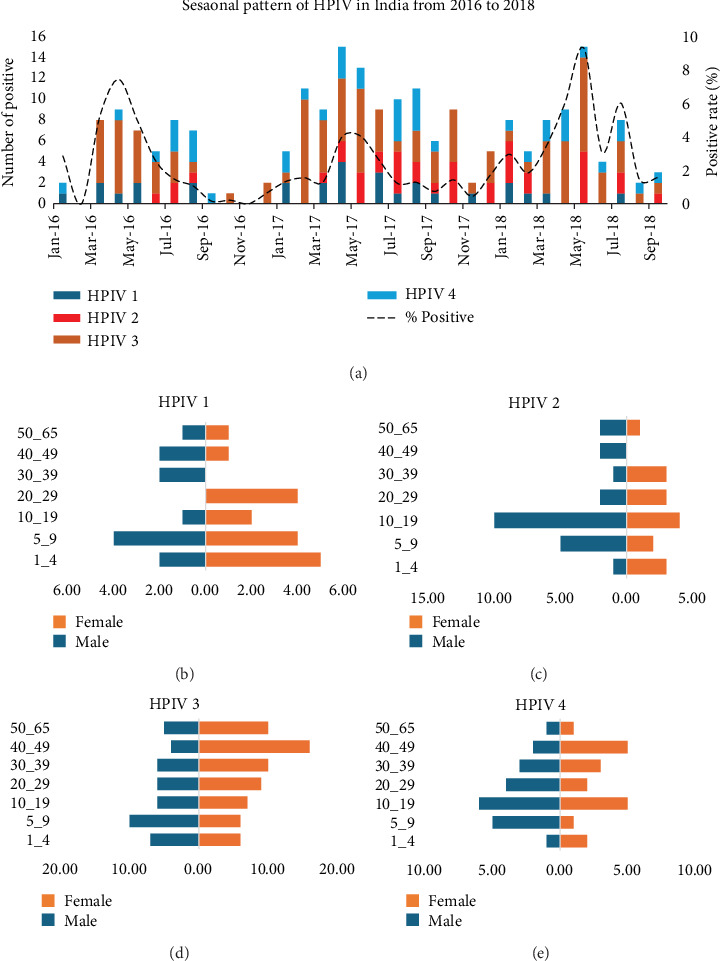
Seasonal trends and age distribution of human parainfluenza virus (HPIV) infections. (a) Seasonality of HPIV serotypes during the period 2016–2018. (b) Age and gender distribution of HPIV 1 cases. (c) Age and gender distribution of HPIV 2 cases. (d) Age and gender distribution of HPIV 3 cases. (e) Age and gender distribution of HPIV 4 cases.

Among the 12,409 patients tested, 217 (1.75%) were positive for HPIVs. Among the positive samples, 29 (13.37%) tested positive for HPIV-1, 39 (17.98%) for HPIV-2, 108 (49.77%) for HPIV-3, and 41 (18.90%) for HPIV-4. The annual incidence of HPIVs varied and was recorded at 1.38% in 2016, 1.61% in 2017, and 3.80% in 2018. The analysis of HPIV seasonality revealed that HPIV-3 presented the greatest number of cases with distinct seasonal peaks, particularly between the late winter and summer seasons in India. HPIV-1, HPIV-2, and HPIV-4 demonstrate sporadic occurrences with lowercase numbers but exhibit seasonal variation (Figure 1a). The age-group distribution indicates that HPIVs predominantly affect younger age groups, particularly children aged 1–9 years, with a relatively balanced sex distribution. HPIV-3 and HPIV-4 cases are more evenly spread across age groups, and HPIV-4 cases are more common among younger individuals, with males being slightly more affected. The incidence of HPIV-2 is greater in adolescents. Overall, HPIV-3 is the most prevalent and seasonally distinct, whereas other types follow less predictable patterns and affect different age groups (Figures 1b, 1c, 1d, and 1e). The samples were represented from 10 states of India, and the percentage positivity and total tested from each state are provided in Table S1.

Among the study participants, upper respiratory tract infection (URTI) symptoms were prevalent, with coryza being the most common (58.9%–80.5%) and sore throat being observed in approximately half of the cases (48.3%–58.5%). LRTI symptoms are frequently reported, with cough being the most common symptom (82.7%–92.7%), followed by shortness of breath (17.2%–31.7%) and chest pain (7.3%–25%). The prevalence of gastrointestinal symptoms varied, with nausea (24.4%–48.3%), vomiting (24.4%–43.5%), abdominal pain (14.8%–43.5%), and diarrhea (1.8%–7.6%) reported in different proportions. Other systemic symptoms, including chills (69.4%–79.3%), myalgia (65.5%–82.9%), arthralgia (44.8%–56.4%), headache (65.5%–82.1%), and general weakness, which was highly prevalent across all serotypes (86.2%–92.6%), were also significant. Conjunctivitis congestion (6.8%–15.3%) and lymphadenopathy (17.6%–27.5%) were less frequently observed (Table 1).

**Figure 2:**
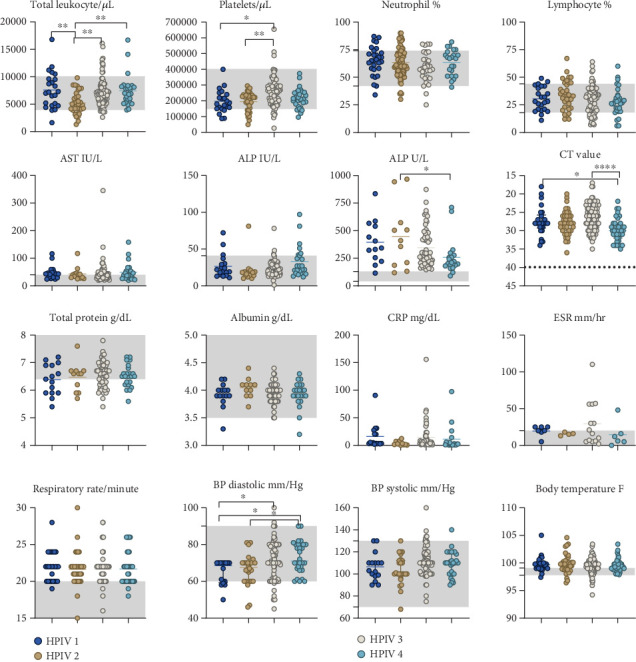
Haematological, biochemical, and clinical parameter variations among patients infected with different human parainfluenza virus (HPIV) types. The line represents median, and the gray band in the graphs indicates normal range (total leukocyte 4000−10,000 cells/μL, platelets 150,000−400,000/μL, neutrophil 45%−75%, lymphocyte 20%−40%, AST 0−40 IU/L, ALT 0−41 IU/L, ALP 40−130 U/L, total protein 6.4−8.3 g/dL, albumin 3.5−5.2 g/dL, CRP 0−10 mg/dL, ESR 20 mm/h, respiratory rate up to 20/min, BP diastolic 60−90 mm/Hg, systolic 70−130 mm/Hg, and body temperature 97°F−99°F). Cycle threshold (CT) represents the viral load, and dotted line indicates the limit of detection by real-time PCR. Statistical significance was determined by one-way ANOVA followed by Tukey's multiple comparisons test (*p* values presented in the figure: < 0.05∗, < 0.01∗∗ and < 0.0001∗∗∗∗). Graphs were made using GraphPad Prism Version 8.4.2.

The findings of this study reveal variations in hematological, biochemical, and inflammatory markers among HPIVs. Leukopenia (< 4000 cells/mm^3^) was present in 3.5%–23.5% of the participants, whereas thrombocytopenia (<150 × 10^3^/*μ*L) was detected in 7.2%–26.4%. The prevalence of neutrophilia (> 70%) ranged from 24.4% to 32.1%, whereas the prevalence of lymphocytosis (> 40%) varied between 11.9% and 30.3%. Liver enzyme abnormalities were notable, with elevated aspartate aminotransferase (AST) (> 40 IU/L) in 39.1%–64.7%, alanine aminotransferase (ALT) (> 40 IU/L) in 6.1%–30.4%, and alkaline phosphatase (ALP) (> 140 IU/L) in 81.8%–98.3%. The erythrocyte sedimentation rate (> 20 mm/h) increased in 20%–50% of the patients. Hypoproteinemia (< 6 g/dL) and hypoalbuminemia (< 3.5 g/dL) were uncommon, reported in 4.3%–33.3% and 0%–6.6% of patients, respectively. Elevated urea (> 40 mg/dL) and creatinine (> 1.2 mg/dL) levels were infrequent. C-reactive protein (> 10 mg/dL) was elevated in 9.1%–37.5% of the participants, indicating variable inflammatory responses (Table 2). Comparative analysis of hematological, biochemical, and physiological parameters among patients with different parainfluenza virus (PIV) types revealed significant variations. Total leukocyte counts were significantly different across types, with HPIV-3 showing lower values. The platelet counts were notably lower in the HPIV-2 group than in the other groups. The percentages of neutrophils and lymphocytes did not significantly differ. Liver function tests revealed variations in ALP levels, with HPIV-3 having significantly higher levels. The CT values were significantly different, indicating variations in the viral load across serotypes. Blood pressure varied significantly, with HPIV-2 showing lower diastolic values. The respiratory rate and body temperature remained relatively consistent across the groups (Figure 2).

**Figure 3:**
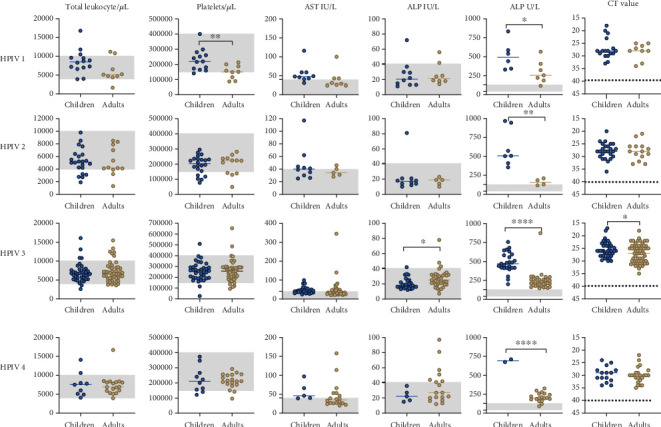
Comparative analysis of hematological, biochemical and viral load parameters between children and adults infected with different HPIV types. The line represents median, and the gray band in the graphs indicates normal range (total leukocyte 4000−10,000 cells/μL, platelets 150,000−400,000/μL, AST 0−40 IU/L, ALT 0−41 IU/L, and ALP 40−130 U/L). Cycle threshold (CT) represents the viral load, and dotted line indicates the limit of detection by real-time PCR. Statistical significance was determined by two tailed *t*-test with 95% confidence interval (*p* values presented in the figure: < 0.05∗, < 0.01∗∗ and < 0.0001∗∗∗∗). Graphs were made using GraphPad Prism Version 8.4.2.

Comparative analysis of hematological and biochemical parameters between children and adults infected with different HPIV types revealed significant variations. Total leukocyte counts remained largely similar across age groups, whereas platelet counts were significantly lower in adults with HPIV-1. Liver enzyme abnormalities were evident, with AST levels being higher in children across all HPIV types. ALT levels were significantly greater in adults with HPIV-3. ALP levels were notably elevated in children across all HPIV types. CT values, reflecting the viral load, were significantly lower in adults with HPIV-3 (Figure 3). Among HPIV cases, the coinfection rate was 3.22% (6), and coinfections with other respiratory viruses were one incidence for each of the following pairs: HPIV-1 and enterovirus, HPIV-2 and respiratory syncytial virus (RSV), HPIV-3 and coronavirus OC43, human metapneumovirus, HPIV-4 and influenza A (H1N1), and influenza B and *Mycoplasma pneumoniae* (Table S1).

## 4. Discussion

The overall incidence of HPIV in this study highlights the distinct seasonal patterns and demographic distributions of HPIVs, with HPIV-3 emerging as the most prevalent type, affecting all age groups. This finding aligns with previous studies that reported HPIV-3 as the dominant strain, peaking in late winter and early summer (7, 8). Our results revealed the notable presence of HPIV-1 and HPIV-2 across different age groups, which is consistent with studies indicating that HPIV-1 contributes primarily to biennial outbreaks in young children, whereas HPIV-2 occurs less frequently but has a broader age distribution (9). The sporadic nature of HPIV-4, which primarily affects younger individuals with a male predominance, is consistent with previous epidemiological data, which suggests that HPIV-4 is less commonly detected but may still contribute to respiratory infections (10).

The clinical presentation observed in our study emphasizes the predominance of respiratory symptoms across all HPIV types, with coryza and cough being the most frequently reported. This finding is also consistent with previous literature describing HPIVs as a major cause of upper and LRTIs (11). The gastrointestinal symptoms noted, particularly nausea, vomiting, and abdominal pain, were more pronounced and significant in certain HPIV types, a trend also observed in studies highlighting the extrarespiratory manifestations of viral infections (12). Systemic symptoms, including myalgia, headache, and weakness, were significantly reported, mirroring findings from other studies that suggest that a generalized inflammatory response contributes to the severity of illness (13). The frequency of conjunctivitis, congestion, and lymphadenopathy was relatively low.

Hematological and biochemical variations among HPIV types were notable, particularly in leukopenia, thrombocytopenia, and liver enzyme abnormalities. Elevated liver enzymes, particularly in HPIV-3 cases, indicate possible hepatic involvement, which has been sporadically reported in other respiratory viruses (14). The differences in CT values suggest variations in viral load, further emphasizing the distinct pathophysiological impacts of different HPIV types. Blood pressure variations, particularly the lower diastolic values observed in HPIV-2 patients, could indicate a differential impact on cardiovascular function, a phenomenon that warrants additional study.

The observed lower platelet counts in adults with HPIV-1 align with previous studies indicating that viral infections can induce transient thrombocytopenia due to immune-mediated platelet depletion or bone marrow suppression (15). Elevated liver enzymes, particularly ALT and AST, in individuals infected with HPIV-3 suggest possible hepatic involvement, either through direct viral effects or immune-mediated injury. In adults, significantly higher ALT levels indicate a distinct hepatocellular response to HPIV-3, which may reflect systemic inflammation. In children, elevated AST across all HPIV types, including HPIV-3, may indicate a broader immune response or mild hepatic stress. Although these enzyme elevations are often transient, they may serve as markers of disease severity. Elevated AST levels in children across all HPIV types are consistent with findings from Weinberg et al., who reported increased liver enzyme activity in pediatric viral respiratory infections (9). Significantly higher ALT levels in adults have been observed in severe viral infections, including influenza and RSV (16). Furthermore, the significantly lower CT values in children with HPIV-3, suggesting a higher viral load, align with research demonstrating that viral replication can be more extensive in children (17). These findings emphasize the importance of considering age-specific variations in HPIV pathophysiology, which could inform altered clinical management strategies.

A significant limitation of this study, and a broader challenge in India, is the lack of a centralized, long-term surveillance system for HPIV, which hinders our ability to establish seasonal patterns, identify high-risk populations, and formulate evidence-based public health interventions. Consequently, the development and implementation of targeted prevention strategies, such as public health campaigns and resource allocation for pediatric care during peak seasons, remain severely underdeveloped. HPIV patients are also coinfected with other respiratory viruses, which might have altered the clinical and laboratory findings. However, these numbers are very low. Data on clinical and laboratory parameters were obtained at the time of admission only. Overall, this study addresses the incidence, distribution, and clinical and laboratory characteristics of HPIV in both children and adults and causes significant morbidity in both children and adults, which requires hospitalization.

## 5. Conclusion

This multiyear surveillance study highlights the incidence of HPIVs in India. Among hospitalized patients with ARI, the overall positivity rate was 1.75%. HPIV-3 was the most prevalent type, showing distinct seasonal peaks from late winter to summer, whereas HPIV-1, HPIV-2, and HPIV-4 occurred sporadically. Children aged 1–9 years were the most affected group, although HPIVs were detected across all age groups. Hematological variations, including leukopenia, thrombocytopenia, and elevated liver enzymes, were common, with HPIV-3 showing a stronger association with hepatic involvement. Age-related differences were evident: children presented higher AST levels and viral loads, whereas adults presented significantly higher ALT levels. Overall, these findings reinforce HPIVs as important contributors to morbidity in both children and adults, frequently leading to hospitalization. This study underscores the need for routine testing, longitudinal monitoring, molecular characterization, and the development of targeted therapeutics to reduce the disease burden associated with HPIVs.

## Figures and Tables

**Table 1:** Comparison of clinical symptoms among patients infected with different human parainfluenza virus (HPIV) types.

**Variables**	**Para 1** **N** ** (%)** **(** **n** = 29**)**	**Para 2** **N** ** (%)** **(** **n** = 39**)**	**Para 3** **N** ** (%)** **(** **n** = 108**)**	**Para 4** **N** ** (%)** **(** **n** = 41**)**	**p** ** value**
**URTI symptoms**					
Coryza	20 (68.9)	23 (58.9)	87 (80.5)	30 (73.2)	0.0621
Sore throat/pharyngitis	14 (48.3)	20 (51.2)	55 (50.9)	24 (58.5)	0.8205
**LRTI symptoms**					
Cough	24 (82.7)	35 (89.7)	100 (92.5)	38 (92.7)	0.4102
Shortness of breath	5 (17.2)	8 (20.5)	22 (20.4)	13 (31.7)	0.4172
Chest pain	5 (17.2)	8 (20.5)	27 (25)	3 (7.3)	0.1121
**Gastrointestinal symptoms**					
Nausea	14 (48.3)	18 (46.1)	30 (27.7)	10 (24.4)	0.0322⁣^∗^
Vomiting	8 (27.5)	17 (43.5)	30 (27.7)	10 (24.4)	0.2230
Abdominal pain	7 (24.1)	17 (43.5)	16 (14.8)	11 (26.8)	0.0036⁣^∗^
Diarrhea	2 (6.8)	3 (7.6)	2 (1.8)	1 (2.4)	0.2830
**Other symptoms**					
Chills	23 (79.3)	28 (71.8)	75 (69.4)	31 (75.6)	0.7098
Myalgia/muscle ache	19 (65.5)	29 (74.3)	77 (71.3)	34 (82.9)	0.3793
Arthralgia/joint pains	13 (44.8)	22 (56.4)	58 (53.7)	19 (46.3)	0.7462
Headache	19 (65.5)	32 (82.1)	84 (77.7)	32 (78.1)	0.3793
General weakness	25 (86.2)	36 (92.3)	100 (92.5)	38 (92.6)	0.7210
Conjunctivitis congestion	2 (6.8)	6 (15.3)	9 (8.3)	5 (12.2)	0.5541
Lymphadenopathy	8 (27.5)	9 (23.1)	19 (17.6)	9 (21.9)	0.6499

*Note:* The statistically significant differences were estimated using chi square test (*p*-values presented in the table: < 0.05⁣^∗^).

**Table 2:** Comparison of haematological and serum chemistry parameters among patients with different human parainfluenza virus (HPIV) types.

**Variables**	**Para 1** **N** ** (%)** **(** **n** = 29**)**	**Para 2** **N** ** (%)** **(** **n** = 39**)**	**Para 3** **N** ** (%)** **(** **n** = 108**)**	**Para 4** **N** ** (%)** **(** **n** = 41**)**	**p** ** value**
Hypotension blood pressure < 60/90 mmHg	1/17 (5.8)	3/26 (11.5)	3/83 (3.6)	1/31 (3.2)	0.9343
Respiratory rate > 24/min	1/26 (3.8)	2/35 (5.7)	7/97 (7.2)	5/36 (13.8)	0.5048
Leukopenia < 4000 cells/mm^3^	2/23 (8.7)	8/34 (23.5)	6/85 (7.1)	1/28 (3.5)	0.0285⁣^∗^
Thrombocytopenia <150 × 10^3^/μL	5/22 (22.7)	9/34 (26.4)	6/83 (7.2)	4/28 (14.3)	0.0330⁣^∗^
Neutrophil percentage > 70%	6/23 (26)	8/33 (24.4)	24/84 (28.5)	9/28 (32.1)	0.0913
Lymphocyte percentage > 40 %	6 /23(26)	10/33 (30.3)	10/84 (11.9)	5/28 (17.8)	0.0949
Aspartate aminotransferase > 40 IU/L	11/17 (64.7)	6/15 (40)	28/65 (43.1)	9/23 (39.1)	0.0034⁣^∗^
Alanine aminotransferase > 40 IU/L	3/17 (17.6)	1/15 (6.6)	4/65 (6.1)	7/23 (30.4)	0.0183⁣^∗^
Alkaline phosphatase > 140 IU/L	12/13 (92.3)	9/11 (81.8)	57/58 (98.3)	18/20(90)	0.1382
Erythrocyte sedimentation rate > 20 mm/h	3/7 (42.8)	1/4 (25)	7/14(50)	1/5 (20)	0.6116
Total protein < 6 g/dL	5/15 (33.3)	3/11 (27.2)	5/59(8.4)	1/23 (4.3)	0.0179⁣^∗^
Albumin < 3.5 g/dL	1/15(6.6)	0/11(0)	1/57 (1.8)	1/23 (4.3)	0.6695
Urea levels > 40 mg/dL	0/16 (0)	1/12(8.3)	3/60 (5.0)	0/23 (0)	—
Creatinine levels > 1.2 mg/dL	0/14 (0)	0/12 (0)	3/60 (5.0)	0/21 (0)	—
C-reactive protein levels > 10 mg/dL	6/16 (37.5)	1/11 (9.1)	17/61(27.8)	4/19(21.1)	0.3806

*Note:* The statistically significant differences were estimated using chi square test (*p*-values presented in the table: < 0.05⁣^∗^).

## Data Availability

The data that support the findings of this study are available from the corresponding author upon reasonable request.

## Supporting Information

Supporting Information Table S1: Additional supporting information can be found online in the Supporting Information section. Demographic characteristics of HPIV infections among patients with ARIs (January 2016–September 2018).
