# Supplementary material for: Distribution and Clinical Profile of Human Parainfluenza Viruses in Hospitalized Patients With Acute Febrile Illness
Source: Int J Microbiol. 2025 Nov 18;2025:7072067. doi: 10.1155/ijm/7072067 (PMC12646732; doi:10.1155/ijm/7072067)
Supplement: Supporting Information Table S1: — Additional supporting information can be found online in the Supporting Information section. Demographic characteristics of HPIV infections among patients with ARIs (January 2016–September 2018). [file 7072067.f1.docx]

***Supplementary Table 1****: Demographic characteristics of HPIVs infections among patients with ARIs (January 2016–September 2018).*

| Variables N (%)  (unless specified) | | Total Tested | HPIVs | HPIV 1 | HPIV 2 | HPIV 3 | HPIV 4 |
| --- | --- | --- | --- | --- | --- | --- | --- |
| State | |  | (n=217) | (n=30) | (n=38) | (n=108) | (n=41) |
|  | Assam | 1258 | 10 (0.8) | 2(0.2) | 1(0.1) | 6(0.5) | 1(0.1) |
|  | Goa | 259 | 3(1.2) | 2 (0.8) |  | 1(0.4) |  |
|  | Gujarat | 458 | 5(1.1) | 1(0.2) | 1(0.2) | 2(0.4) | 1(0.2) |
|  | Jharkhand | 381 | 23 (6.3) | 7(1.8) | 2(0.5) | 10(2.6) | 4(1.1) |
|  | Karnataka | 3496 | 84(2.4) | 6((0.2) | 11(0.3) | 52(1.5) | 15(0.4) |
|  | Kerala | 1026 | 12(1.2) | 1(0.1) | 2(0.8) | 5(0.5) | 4(0.4) |
|  | Maharashtra | 379 | 8(2.1) | 1(0.3) | 3(0.8) | 4(1.1) |  |
|  | Odisha | 490 | 12(2.4) | 1(0.2) | 3(0.6) | 7(1.4) | 1(0.2) |
|  | Tamil Nadu | 3593 | 58(1.6) | 7(0.2) | 15(0.4) | 21(0.6) | 15(0.4) |
|  | Tripura | 781 | 2(0.3) | 2(0.3) |  |  |  |
| Age in years | |  | 217 | n=29 | n=39 | n=108 | n=31 |
|  | 1 to 4 | 572 | 27 (4.7) | 7(1.2) | 4(0.7) | 13(2.3) | 3(0.5) |
|  | 5 to 9 | 1659 | 27(1.6) | 8(0.5) | 7(0.4) | 16(0.9) | 6(0.4) |
|  | 10 to 19 | 2848 | 41(1.4) | 3(0.1) | 14(0.5) | 13(0.4) | 11(0.4) |
|  | 20 to 29 | 2266 | 30(1.3) | 4(0.2) | 5(0.2) | 15(0.7) | 6(0.3) |
|  | 30 to 39 | 1808 | 28(1.5) | 2(0.1) | 4(0.2) | 16(0.9) | 6(0.3) |
|  | 40 to 49 | 1545 | 32(2.1) | 3(0.2) | 2(0.1) | 20(1.3) | 7(0.5) |
|  | 50 to 65 | 1417 | 22(1.6) | 2(0.2) | 3(0.2) | 15(1.1) | 2(0.1) |
| Gender | |  |  | n=28 | n=39 | n=109 | n=41 |
|  | Male | 6127 | 101(1.6) | 12(0.2) | 23(0.4) | 44(0.7) | 22(0.4) |
|  | Female | 5993 | 116(1.9) | 16(0.3) | 16(0.3) | 65(1.1) | 19(0.3) |
| Co-infection with HPIVs | |  | 7(3.2) | 1(0.5) | 1(0.5) | 2(0.9) | 3(1.4) |
|  | HCoV-OC 43 | 217 | 1(0.5) |  |  | 1(0.5) |  |
|  | Enterovirus | 217 | 1(0.5) | 1(0.5) |  |  |  |
|  | HMPV | 217 | 1(0.5) |  |  | 1(0.5) |  |
|  | Influenza A H1N1 | 217 | 1(0.5) |  |  |  | 1(0.5) |
|  | Influenza B | 217 | 1(0.5) |  |  |  | 1(0.5) |
|  | RSV | 217 | 1(0.5) |  | 1(0.5) |  |  |
|  | M Pneumoniae | 217 | 1(0.5) |  |  |  | 1(0.5) |
